# Allelic combinations of *Hd1*, *Hd16*, and *Ghd7* exhibit pleiotropic effects on agronomic traits in rice

**DOI:** 10.1093/g3journal/jkad300

**Published:** 2024-01-03

**Authors:** Seung Young Lee, Ji-Ung Jeung, Youngjun Mo

**Affiliations:** Department of Crop Science and Biotechnology, Jeonbuk National University, Jeonju 54896, Republic of Korea; National Institute of Crop Science, Rural Development Administration, Wanju 55365, Republic of Korea; National Institute of Crop Science, Rural Development Administration, Wanju 55365, Republic of Korea; Department of Crop Science and Biotechnology, Jeonbuk National University, Jeonju 54896, Republic of Korea

**Keywords:** Plant Genetics and Genomics, rice, QTL, *Hd1*, *Hd16*, *Ghd7*, allelic combination

## Abstract

Heading date is a critical agronomic trait that significantly affects grain yield and quality in rice. As early heading is typically associated with reduced yield due to shorter growth duration, it is essential to harness optimum heading date genes and their allelic combinations to promote heading while minimizing yield penalties. In this study, we identified quantitative trait loci (QTLs) for heading date and other major agronomic traits in a recombinant inbred line (RIL) population derived from a cross between Koshihikari and Baegilmi. Analyses on 3 major QTLs for heading date and their underlying genes (*Hd1*, *Hd16*, and *Ghd7*) revealed their pleiotropic effects on culm length, panicle length, and head rice percentage. Additionally, *Ghd7* exhibited pleiotropic effects on panicle number and grain size. Among 8 different types of allelic combinations of the 3 heading date genes, RILs carrying a single nonfunctional *hd16* or *ghd7* under the functional background of the other 2 genes (*Hd1hd16Ghd7* and *Hd1Hd16ghd7*) showed potential for maintaining yield and quality-related traits while accelerating heading. These results provide valuable insights for fine-tuning heading dates in rice breeding programs.

## Introduction

Flowering time, also known as the heading date, is a critical agronomic trait determining crop productivity (Izawa 2007a; Cho *et al*. 2017). Environmental factors such as day length and temperature exert a significant influence on the heading date, interacting with inherent characteristics such as photoperiod sensitivity, temperature sensitivity, and vegetative growth duration (Shang *et al*. 2012; Shrestha *et al*. 2014; Itoh *et al*. 2018). Either extremely early or overly late flowering can result in reduced grain yield attributed to insufficient sink strength or the susceptibility to adverse weather conditions during grain filling (Jung and Müller 2009; Gao *et al*. 2014; Li *et al*. 2018). Therefore, to optimize grain yield, it is essential to develop varieties with heading dates tailored for specific target environments. This optimized timing enables the maximization of available light and temperature resources within their respective cultivating regions, ensuring successful production.

To finely tune heading dates, it is crucial to understand and utilize the genetic mechanisms underlying heading date variations. In rice, numerous quantitative trait loci (QTLs) associated with heading date have been identified through genetic approaches (Yamamoto *et al*. 2012; Hori *et al*. 2016; Yano *et al*. 2016; Matsubara and Yano 2018). Furthermore, molecular investigations into the genes influencing heading date have unveiled their pleiotropic effects on various agronomic traits. For instance, *Heading date 1* (*Hd1*) (Yano *et al*. 2000) is a component of *OsGI*-*Hd1*-*Hd3a* (rice *GIGANTEA*, *Heading date 1*, and *Heading date 3a*) signaling pathway. This pathway exhibits evolutionary conservation analogous to the *GI*-*CO*-*FT* (*GIGANTEA*, *CONSTANS*, and *FLOWERING LOCUS T*) pathway of *Arabidopsis*. Within this regulatory network, the upregulation of *Hd1*, mediated by *OsGI*, triggers the activation of *Hd3a* expression, thus inducing rice heading under both short-day and long-day conditions. The combination of *Hd1* and *Early heading date 1* (*Ehd1*) regulates panicle development, potentially impacting crop yield (Endo-Higashi and Izawa 2011). Additionally, delayed heading under long-day conditions influenced by *Hd1* and *Grain number, plant height and heading date 7* (*Ghd7*) interaction, contributes to enhanced plant height and higher grain yield (Yano *et al*. 2001; Hayama *et al*. 2003; Searle and Coupland 2004; Nemoto *et al*. 2016; Zhang *et al*. 2017). The other pathway involves the rice-specific gene *Ghd7*, which encodes CO, CO-LIKE, and TOC1 (CCT) domain proteins and has been identified as a flowering repressor under long-day conditions due to its inhibitory effect on *Hd3a* (Xue *et al*. 2008). Moreover, *Ghd7* hinders the expression of *Ehd1*, an upstream regulator of *Hd3a* (Doi *et al*. 2004) and plays a pivotal role in regulating plant height, heading date, and yield (Xue *et al*. 2008). The *Heading date 16* (*Hd16*) encodes casein kinase I, a key regulator in the photoperiodic flowering pathway of rice. *Hd16* exerts its influence by initiating the activation of *Ghd7* through phosphorylation. Within this framework, a missense mutation occurring in exon 10 leads to a reduction in kinase activity, resulting in the acceleration of heading under long-day conditions (Hori *et al*. 2013).

Previously, we performed QTL analysis on rice heading date using recombinant inbred lines (RILs) derived from 2 rice cultivars, Koshihikari and Baegilmi, and revealed that *Hd1*, *Hd16*, and *Ghd7* underlie the 3 major heading date QTLs (Mo *et al*. 2020). In the present study, we used the same RIL population to conduct QTL analysis on major agronomic traits and characterize the pleiotropic effects and interactions of *Hd1*, *Hd16*, and *Ghd7*. By analyzing the RILs with different allelic combinations of these 3 genes, we also defined potentially advantageous allelic combinations that could accelerate heading while maintaining grain yield and quality-related traits, providing valuable insights for rice breeding programs.

## Materials and methods

### Plant materials and field test conditions

A total of 339 RILs derived from a cross between 2 *japonica* rice varieties, Koshihikari and Baegilmi, were used in this study. For QTL analysis, a subset (*n* = 142) of the RILs was used while the whole population (*n* = 339) were utilized to investigate the effects of 3 heading date genes, *Hd1*, *Hd16*, and *Ghd7*, on agronomic traits (Supplementary Tables 1–3). All plant materials were cultivated in the experimental field of the National Institute of Crop Science (NICS), Wanju, South Korea (35°84′N 127°05′E). Field tests (FTs) were conducted from May to October in 2016 (F_6_; FT 1), 2017 (F_7_; FT 2), and 2018 (F_8_; FT 3). Climate conditions during the FTs are shown in Supplementary Table 4. The seedlings were transplanted into the paddy field 4 weeks after sowing under the following conditions: 1 RIL per row, 30 plants per row, and 1 plant per hill, with a spacing of 15 cm between plants in a row and 30 cm between adjacent rows. Fertilizers were applied uniformly across all FTs, with a dosage of 9 kg N/10a, 4.5 kg P_2_O_5_/10a, and 4.5 kg K_2_O/10a. The plants were grown and managed according to the standard rice cultivation methods of NICS, Rural Development Administration (RDA) (RDA 2012).

### Phenotype evaluation

Days to heading (DTH) of each RIL were determined by counting the number of days from sowing to heading when the panicles of 40% of plants emerged in a row. Agronomic traits were collected from 10 randomly chosen plants in each line across 3 FTs. Culm length (CL) was measured as the length from the ground level to the panicle node of the main culm. Panicle length (PL) was determined by measuring the length from the panicle node to the end of the main panicle. The panicle number per plant (PN) was calculated by counting the total number of panicles per plant. The grain characteristics of brown rice were assessed only during FT2. Each RIL was harvested 50–55 days after heading upon maturity, dried, and maintained at 15% moisture content in a storage room at 10°C. After the entire RIL population was harvested, the samples were dehulled to measure the grain characteristics of brown rice. The head rice percentage (HRP) was determined using RN300 Rice Quality Analyzer (Kett, Japan) with the “Approval” mode of Quality Scan software for brown rice, which classified the grain based on 3 criteria: even, cracked, and others (immature, dead, and discolored). The percentage of grains classified as “even” over total grains was calculated to generate HRP. Thousand-grain weight (TGW) was measured by determining the weight of 1,000 dehulled grains. Grain length (GL) and grain width (GW) of each RIL were measured using 10 randomly selected fully filled grains with a Vernier caliper.

### QTL analysis and allele determination of heading date genes

Genomic DNA was extracted from the young leaves of each RIL using the modified cetyltrimethylammonium bromide method (Murray and Thompson 1980). The concentration and quality of each DNA sample were assessed using the DeNovix DS-11 spectrophotometer (DeNovix, USA). A total of 128 high-quality polymorphic SNPs from the 142 RILs (missing rate < 10%) previously described in Mo *et al*. (2020) were used for linkage map construction. The QTL analysis was conducted using QTL ICIMapping 4.2 (Meng *et al*. 2015). The Kosambi mapping function was used to calculate recombination distance (Kosambi 1944). The inclusive composite interval mapping of additive (ICIM-ADD) method was used to detect QTLs with the default mapping parameters (step cM as 1.0 and PIN as 0.001). The logarithm of the odds (LOD) threshold for declaring significant QTLs ranged from 2.6 to 3.1 according to 1,000 permutation tests at *P* = 0.05. Phenotypic variance explained (PVE) and additive effect (ADD) estimates of each QTL position were obtained from the ICIM-ADD analysis.

Molecular markers used for genotyping *Hd1*, *Hd16*, and *Ghd7* in the 339 RILs are described in Mo *et al*. (2020). Each PCR reaction comprised 20 ng of template DNA, 5 pmol of each primer, 10 mM of dNTPs, 10× PCR buffer, and 0.5 U of *Taq* DNA polymerase (Inclone). For *Hd1* and *Ghd7* genotyping, DNA was initially incubated for 5 min at 94°C followed by 35 cycles of amplification: denaturation at 94°C for 30 s, annealing at the proper melting temperature (58°C for *Hd1* and 60°C for *Ghd7*) for 30 s, and extension at 72°C for the relevant duration (30 s for *Hd1* and 240 s for *Ghd7*) followed by final extension at 72°C for 10 min. For *Hd16*, PCR was conducted by a combined procedure of annealing and extension steps at 68°C for 60 min, followed by the *NheI* digestion of the PCR product. Detailed primer sequences and expected band patterns are shown in Supplementary Table 5.

### Statistical analysis

All statistical analyses were performed using R version 4.2.1 R Core Team (2022). The frequency distribution and correlation plots of the agronomic traits among different FTs were analyzed using “ggpubr” package. To examine the interactions and allelic combination effects among *Hd1*, *Hd16*, and *Ghd7*, we utilized the mean values of agronomic traits across the 3 FTs. Boxplots visualizing the agronomic traits and grain characteristics of different allelic combinations were created using “ggplot2”, “gridExtra”, “gapminder”, and “dplyr” packages. The mean comparison of traits among different allelic combinations was conducted by Duncan's multiple range test using “agricolae” package (Mendiburu and Yaseen 2020). To verify the existence of high-order genetic interactions, a 3-way factorial ANOVA was performed to determine the main effects of 3 heading date genes using “rstatix”, “lme4”, “lmertest”, and “car” packages. To visualize the simple effects of the heading date genes, 2-way and 3-way interaction plots were generated using “dplyr” package. Correlation analyses and their visualization were conducted using Pearson's correlation coefficient (*P* ≤ 0.05) via the “corrplot” package.

## Results

### Variation of major agronomic traits in the Koshihikari/Baegilmi RIL population

**Fig. 1.**
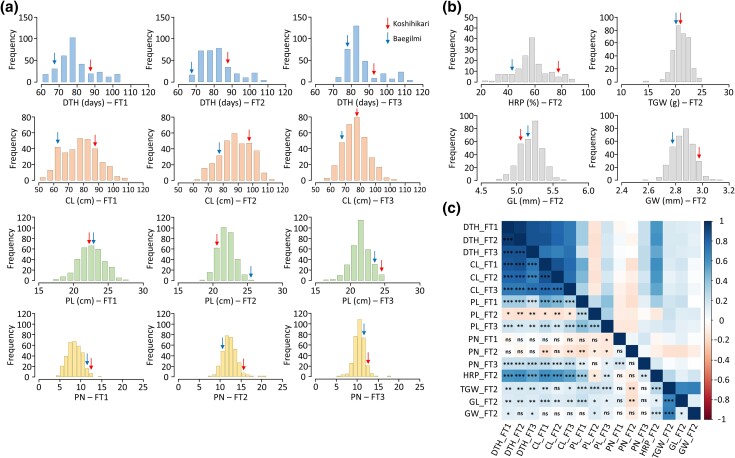
The frequency distribution of 8 agronomic traits in the Koshihikari/Baegilimi RIL population. a) Variations in the DTH, CL, PL, and PN in field test 1 (FT1), field test 2 (FT2), and field test 3 (FT3). b) Variation in the HRP, TGW, GL, and GW in FT2. Arrows indicate phenotypic values of the parents, Koshihikari and Baegilmi. c) Correlations among 8 agronomic traits in all FTs. Pearson's correlation coefficient (*r*) was employed to estimate the strength of correlation among FTs. *P < 0.05; **P < 0.01; ***P < 0.001; ns, not significant.

The phenotypic frequency distribution of 8 agronomic traits among the 339 RILs derived from a cross between Koshihikari and Baegilmi are shown in Fig. 1a and b. The traits CL, PL, PN, HRP, TGW, GL, and GW were normally distributed, whereas DTH showed a rightly skewed distribution. In the 3 FTs, the DTH of Koshihikari ranged from 86 to 91 days, whereas those of Baegilmi ranged from 67 to 78 days. The DTH values of the 339 RILs varied from 64 to 113 days across the 3 FTs. In addition, the RIL population showed large phenotypic variation in other agronomic traits, with ranges of 51.3–114 cm for CL, 16.5–27.3 cm for PL, 4.5–20.3 for PN, 21.6–89.7% for HRP, 14.5–25.0 g for TGW, 4.7–5.6 mm for GL, and 2.6–3.1 mm for GW. Transgressive segregations were observed in all traits, as a number of RILs displayed agronomic trait values higher or lower than both parents. Strong positive correlations were observed among the DTH values of the RILs for all 3 FTs, with correlation coefficients of 0.86–0.95 (Fig. 1c). Additionally, CL and PL showed significant correlations among the FTs, whereas PN exhibited significant correlations only between FT1 and FT3.

### QTL analysis of the agronomic traits of RILs

**Fig. 2.**
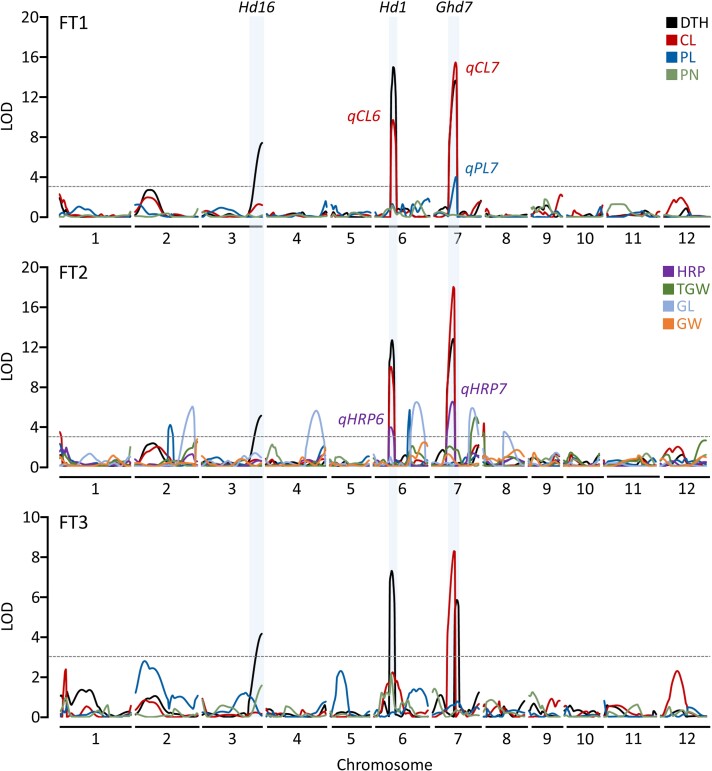
QTL mapping for agronomic traits from the Koshihikari/Baegilimi RILs. The horizontal dashed line denotes the LOD score of 3.0 (LOD thresholds ranged from 2.6 to 3.1 according to 1,000 permutation tests at *P* = 0.05). Shaded regions indicate the locations of previously detected heading date genes, i.e. *Hd16*, *Hd1*, and *Ghd7*.

A total of 128 SNPs from a subset (*n* = 142) of the RILs were used to construct a linkage map encompassing 1,293 cM composed of an average of 10.7 markers per chromosome, with an average genetic distance of 11.1 cM between adjacent markers (Mo *et al*. 2020). Three significant QTLs were detected for DTH in 3 FTs (Fig. 2 and Table 1). These 3 stable QTLs harbored previously identified heading date genes, *Hd1* (*qDTH6*), *Hd16* (*qDTH3*), and *Ghd7* (*qDTH7*) (Mo *et al*. 2020). *qDTH6* colocalized with *qCL6* in 2 FTs and *qHRP6* in 1 FT. *qDTH7* colocalized with *qCL7* in 3 FTs, *qPL7* in 1 FT, and *qHRP7* in 1 FT. These results confirmed the previously well-known pleiotropic effects of *Hd1* and *Ghd7* on multiple agronomic traits (Xue *et al*. 2008; Zhang *et al*. 2012; Nemoto *et al*. 2016).

**Table 1.** Summary of QTLs for agronomic traits from the Koshihikari/Baegilimi RILs.

Trait	Field test	QTL	Chr.*^a^*	Flanking markers*^b^*		LOD*^c^*	PVE*^d^* (%)	Add*^e^*
				Left	Right			
DTH	1	*qDTH3*	3	S3_28142709	S3_34851991	7.5	11.2	−3.5
	1	*qDTH6*	6	S6_8634012	S6_10449013	15.0	24.6	5.2
	1	*qDTH7*	7	S7_5256691	S7_10453336	13.7	22.2	4.9
	2	*qDTH3*	3	S3_28142709	S3_34851991	5.1	8.9	−3.2
	2	*qDTH6*	6	S6_8634012	S6_10449013	12.6	25.9	5.4
	2	*qDTH7*	7	S7_5256691	S7_10453336	12.7	24.9	5.3
	3	*qDTH3*	3	S3_28142709	S3_34851991	4.2	9.2	−2.9
	3	*qDTH6*	6	S6_8634012	S6_10449013	7.3	17.3	4.0
	3	*qDTH7*	7	S7_14589984	S7_17610718	5.8	13.3	3.5
CL	1	*qCL6*	6	S6_8634012	S6_10449013	9.7	19.1	5.1
	1	*qCL7*	7	S7_5256691	S7_10453336	15.5	33.4	6.7
	2	*qCL1*	1	S1_2279203	S1_3915956	3.4	4.7	2.4
	2	*qCL6*	6	S6_8634012	S6_10449013	9.9	16.8	4.5
	2	*qCL7*	7	S7_5256691	S7_10453336	18.0	34.7	6.4
	2	*qCL8*	8	S8_633037	S8_1149395	4.2	6.2	−2.7
	3	*qCL7*	7	S7_5256691	S7_10453336	8.3	23.5	3.9
PL	1	*qPL7*	7	S7_5256691	S7_10453336	4.0	13.3	0.6
	2	*qPL2*	2	S2_22347808	S2_24136482	4.2	11.2	−0.5
	2	*qPL6*	6	S6_22698235	S6_23237994	5.6	13.8	−0.5
HRP	2	*qHRP6*	6	S6_8634012	S6_10449013	3.9	10.2	4.7
	2	*qHRP7*	7	S7_5256691	S7_10453336	6.5	19.9	6.5
TGW	2	*qTGW7*	7	S7_22937275	S7_26676166	5.0	14.8	0.6
	2	*qTGW8*	8	S8_133918	S8_633037	3.4	6.9	0.4
GL	2	*qGL2*	2	S2_25869013	S2_32050487	6.0	11.1	0.1
	2	*qGL4*	4	S4_27737927	S4_31439312	5.6	10.5	−0.1
	2	*qGL6*	6	S6_25507580	S6_2807760	6.4	12.2	−0.1
	2	*qGL7*	7	S7_22937275	S7_26676166	5.8	11.8	0.1
	2	*qGL8*	8	S8_10026296	S8_24214130	3.5	6.3	0.0

^*a*^Chromosome number.

^*b*^The number after the “S” indicates the chromosome number and physical position of SNP according to the IRGSP-1.0 reference genome.

^*c*^Logarithm of the odds. LOD thresholds ranged from 2.6 to 3.1 according to 1,000 permutation tests at *P* = 0.05.

^*d*^Phenotypic variation explained.

^*e*^Positive additive effect (Add) indicates that Koshihikari allele contributes positively to the trait.

### Effects of *Hd1*, *Hd16*, and *Ghd7* on major agronomic traits

**Fig. 3.**
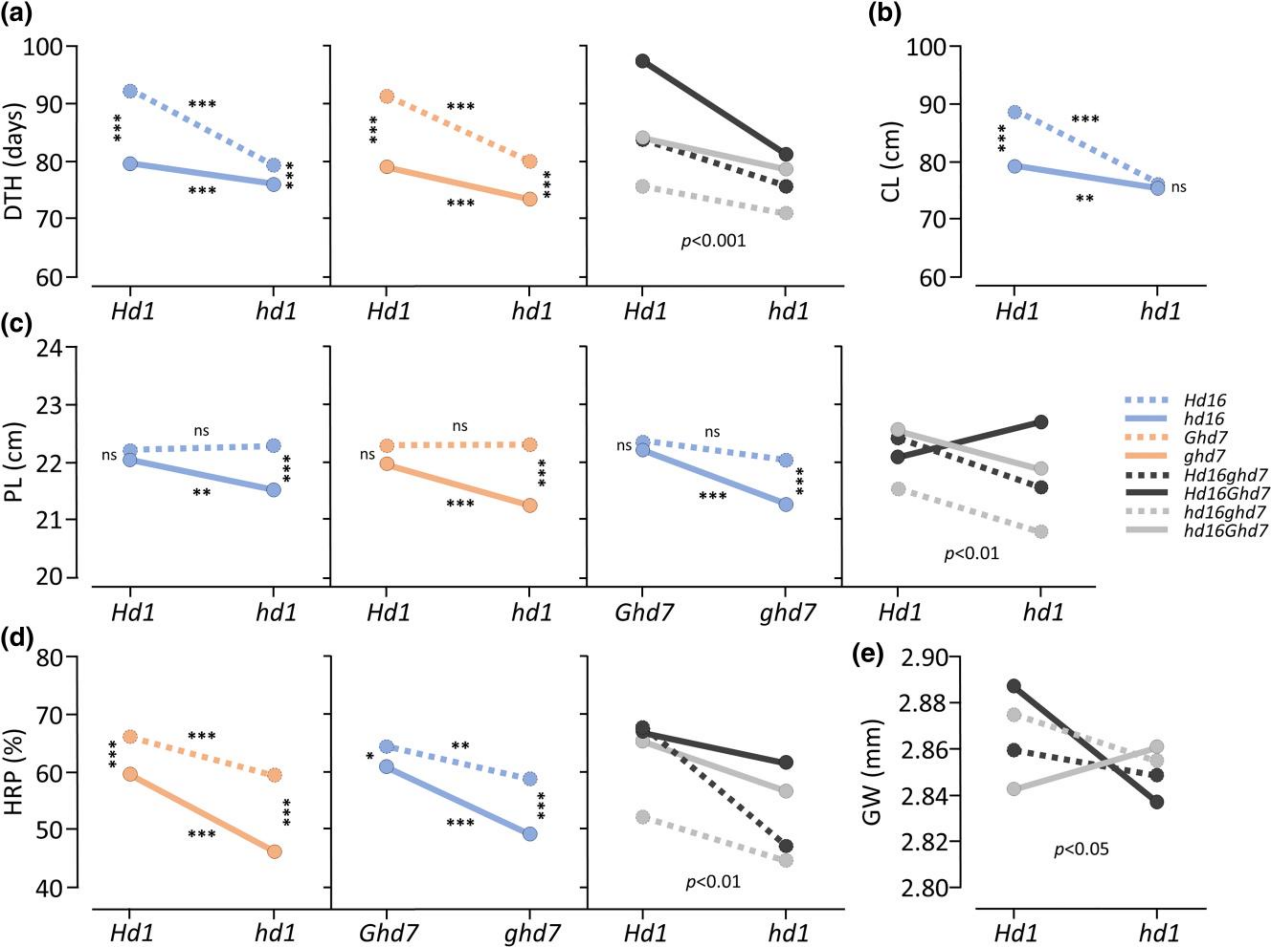
Interaction plots of allelic effects of *Hd1*, *Hd16*, and *Ghd7* on major agronomic traits. a) 2-way and 3-way interactions on DTH. b) *Hd1×Hd16* interaction on CL. c) 2-way and 3-way interactions on PL. d) 2-way and 3-way interactions on HRP. e) 3-way interaction on GW. Only the significant interactions are plotted. *P < 0.05. **P < 0.01. ***P < 0.001. ns, not significant.

Previous studies confirmed that Koshihikari carries a G-to-A (alanine-to-threonine) mutation of *Hd16* responsible for early heading under long day (hereafter *hd16*) and functional alleles of *Hd1* and *Ghd7*, while Baegilmi carries nonfunctional *Hd1* due to a 43-bp deletion in exon 2 (hereafter *hd1*) and a 1901-bp retrotransposon insertion in the promoter region of *Ghd7* (hereafter *ghd7*) with Nipponbare-type functional *Hd16* (Yano *et al*. 2000; Hori *et al*. 2013; Fujino and Yamanouchi 2020; Mo *et al*. 2020). Using a larger number (*n* = 339) of RILs, we characterized the main effects of the 3 heading date genes (*Hd1*, *Hd16*, and *Ghd7*) and their 2-way and 3-way interactions on major agronomic traits (Table 2, Fig. 3).

**Table 2.** Three-way ANOVA of *Hd1*, *Hd16*, and *Ghd7* on the agronomic traits.

Trait	Value*^a^*	Main effect			Interaction			
		*Hd1* (A)	*Hd16* (B)	*Ghd7* (C)	A × B	A × C	B × C	A × B × C
DTH (days)	Allele a	86.1	78.2	86.0				
	Allele b	77.8	86.4	77.0				
	PVE (%)	23.9	17.9	26.0	4.2	1.6	0.2	1.2
	*P*-value	***	***	***	***	***	ns	***
CL (cm)	Allele a	84.4	77.8	84.8				
	Allele b	76.1	83.4	74.5				
	PVE (%)	22.7	6.7	29.9	3.6	0.0	0.2	0.2
	*P*-value	***	***	***	***	ns	ns	ns
PL (cm)	Allele a	22.1	21.8	22.3				
	Allele b	21.9	22.3	21.7				
	PVE (%)	3.1	4.5	9.3	1.4	2.6	1.9	1.9
	*P*-value	***	***	***	*	***	**	**
PN (no. per plant)	Allele a	10.5	10.5	10.6				
	Allele b	10.4	10.4	10.3				
	PVE (%)	0.3	0.3	1.6	0.5	0.0	0.3	0.8
	*P*-value	ns	ns	*	ns	ns	ns	ns
HRP (%)	Allele a	63.4	56.5	62.9				
	Allele b	54.8	62.4	54.2				
	PVE (%)	13.7	4.9	11.9	0.7	1.7	1.2	2.1
	*P*-value	***	***	***	ns	**	*	**
TGW (g)	Allele a	21.1	21.0	21.3				
	Allele b	21.1	21.2	20.8				
	PVE (%)	0.0	0.1	2.8	1.5	0.1	0.1	0.0
	*P*-value	ns	ns	*	ns	ns	ns	ns
GL (mm)	Allele a	5.2	5.2	5.2				
	Allele b	5.2	5.2	5.1				
	PVE (%)	0.0	0.0	3.8	1.3	0.0	0.0	0.0
	*P*-value	ns	ns	***	ns	ns	ns	ns
GW (mm)	Allele a	2.9	2.9	2.8				
	Allele b	2.9	2.9	2.8				
	PVE (%)	1.0	0.0	0.0	1.0	0.0	0.5	1.5
	*P*-value	ns	ns	ns	ns	ns	ns	*

For DTH, CL, PL, and PN which were evaluated under 3 field tests (FTs), 3-way ANOVAs were conducted with the 3 genes as fixed variables and the FTs as random variables.

*P < 0.05; **P < 0.01; ***P < 0.001; ns, not significant.

^*a*^Allele a and b represent Koshihikari and Baegilmi allele, respectively. PVE refers to the proportion of the total phenotypic variance of a trait attributed to each genetic component.

#### Days to heading

The main effects of *Hd1*, *Hd16*, and *Ghd7* were highly significant, explaining 23.9, 17.9, and 26.0% of the DTH variation, respectively (Table 1). On average, RILs carrying *ghd7* (Baegilmi allele) headed 9.0 days earlier than those carrying *Ghd7* (Koshihikari allele). Similarly, *hd1* (Baegilmi allele) and *hd16* (Koshihikari allele) accelerated heading by 8.3 and 8.2 days, respectively. The 2-way and 3-way interactions among *Hd1*, *Hd16*, and *Ghd7* were highly significant (*P* < 0.001) except for the *Hd16×Ghd7* interaction (Fig. 3a). In both the *Hd1×Hd16* and the *Hd1×Ghd7* interactions, the acceleration of DTH by the nonfunctional allele of 1 gene exhibited a greater effect when coexisting with the functional allele of another gene. For example, *hd1* accelerated heading by 13.0 days and 3.6 days under the background of *Hd16* and *hd16*, respectively. Similarly, *hd1* allele accelerated heading by 11.3 days and 5.5 days when combined with *Ghd7* and *ghd7*, respectively. The 3-way (*Hd1×Hd16×Ghd7*) interaction indicated that the effect of the nonfunctional allele of 1 gene is the greatest in the presence of the functional alleles of the other 2 genes.

#### Culm length

Significant differences in CL (*P* < 0.001) were observed for all 3 heading date genes, with the nonfunctional alleles being associated with shorter CL. *Ghd7* explained the largest proportion (29.9%) of CL variation, exhibiting a 10.3 cm difference between the RILs carrying the functional and nonfunctional alleles, followed by *Hd1* (22.7%, 8.3 cm) and *Hd16* (6.7%, 5.6 cm). However, only the *Hd1×Hd16* interaction was significant (Fig. 3b), with *hd1* decreasing CL by 12.6 and 3.8 cm under the background of *Hd16* and *hd16*, respectively.

#### Panicle length

The main effects of the 3 heading date genes and their interaction effects were highly significant (*P* < 0.001), accounting for 24.7% of the total PL variation. Similar to CL, the nonfunctional allele of each gene was associated with shorter PL, with *Ghd7* being the largest contributor (9.3%, 0.6 cm), followed by *Hd16* (4.5%, 0.5 cm) and *Hd1* (3.1%, 0.2 cm). Interestingly, the patterns of the 2-way and 3-way interactions on PL were different from those on DTH and CL. For example, the *Hd1×Hd16* interaction showed that *hd1* decreased PL by 0.5 cm under the *hd16* background while exhibiting no significant effect under the *Hd16* background (Fig. 3c). Similarly, the *Hd1×Ghd7* and the *Hd16×Ghd7* interactions also indicated that the effect of the nonfunctional allele of 1 gene on decreasing PL is significant only under the presence of the nonfunctional allele of another gene. The 3-way (*Hd1×Hd16×Ghd7*) interaction showed that *hd1* increased PL under the presence of both *Hd16* and *Ghd7*, while decreasing PL under the presence of either *hd16* or *ghd7*.

#### Panicle number

Only the main effect of *Ghd7* was marginally significant (*P* < 0.05), explaining 1.6% of the PN variation. There was no significant 2-way or 3-way interaction of the 3 heading date genes on PN.

#### Head rice percentage

The main effects and interactions of the 3 heading date genes accounted for 36.2% of the HRP variation, with the nonfunctional allele of each gene associated with lower HRP. On average, the RILs carrying *hd1*, *hd16*, and *ghd7* exhibited 8.6, 5.9, and 8.7% lower HRP compared to those carrying *Hd1*, *Hd16*, and *Ghd7*, respectively. Notably, all 2-way and 3-way interactions except the *Hd1×Hd16* interaction were significant (Fig. 3d). The *hd1* allele decreased HRP by 6.7 and 13.1% under the background of *Ghd7* and *ghd7*, respectively. Similarly, *ghd7* allele decreased HRP by 5.6 and 11.6% when combined with *Hd16* and *hd16*, respectively. Interestingly, the 3-way interaction revealed that the effect of the nonfunctional allele of 1 gene decreasing HRP can be mitigated under the presence of the functional alleles of the other 2 genes.

#### Thousand-grain weight and grain length

Only the main effect of *Ghd7* was significant, with the nonfunctional *ghd7* allele associated with reduced TGW and GL. There were no significant 2-way or 3-way interactions of the 3 heading date genes on TGW and GL.

#### Grain width

The 3 heading date genes and their interaction exhibited minimal effects on GW, with only the 3-way interaction being marginally significant (*P* < 0.05) explaining 1.5% of the GW variation (Fig. 3e).

### Effects of the allelic combinations of *Hd1*, *Hd16*, and *Ghd7* on major agronomic traits

**Fig. 4.**
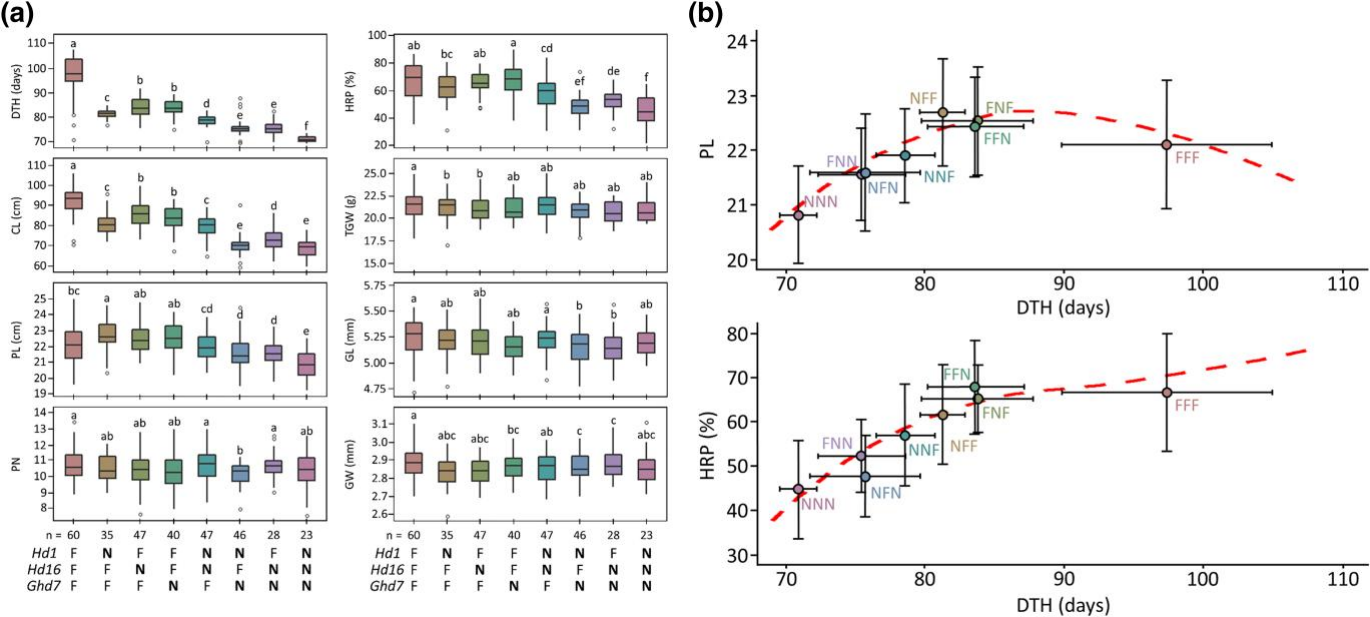
Effects of the allelic combinations of *Hd1*, *Hd16*, and *Ghd7* on major agronomic traits. a) Combined boxplots of each agronomic trait according to the 8 allelic combinations. The different letters above the boxplots denote significance among allele combinations at *P* < 0.05 determined by Duncan's multiple range test. F and N indicate functional and nonfunctional allele, respectively. Bold letter indicates allele associated with early heading. b) The relationship among DTH, PL, and HRP. Error bar and dashed line represent standard deviation and LOESS (locally estimated scatterplot smoothing), respectively.

To evaluate the effects of pyramiding nonfunctional *hd1*, *hd16*, and *ghd7* alleles on major agronomic traits, we compared the agronomic traits of the RILs carrying 8 different allelic combinations of the 3 genes (Fig. 4a). Compared to the RILs carrying *Hd1Hd16Ghd7* (FFF; F refers to a functional allele), those with a single nonfunctional allele, i.e. *hd1Hd16Ghd7* (NFF; N refers to a nonfunctional allele), *Hd1hd16Ghd7* (FNF), and *Hd1Hd16ghd7* (FFN), exhibited significantly earlier DTH and shorter CL. The RILs with the NFF combination exhibited greater reduction in DTH and CL compared to those with the FNF and FFN combinations, indicating a stronger effect of *hd1* compared to *hd16* and *ghd7*. However, there was no significant difference in PL, PN, TGW, GL, and GW among the RILs carrying the NFF, FNF, and FFN combinations. When 2 nonfunctional alleles were pyramided, i.e. *hd1hd16Ghd7* (NNF), *hd1Hd16ghd7* (NFN), and *Hd1hd16ghd7* (FNN), substantial reductions were observed in DTH, CL, PL, and HRP compared to the impact of a single nonfunctional allele. Furthermore, pyramiding the 3 nonfunctional alleles (NNN) showed the most significant decreases in the aforementioned traits.

Next, we focused on PL and HRP to test the possibility of identifying optimal allele combinations with reduced growth duration while concurrently preserving agronomic traits affecting yield and grain quality (Fig. 4b). PL increased with an increase in DTH, but reached a limit at 88 DTH. Notably, the allele combinations of NFF, FNF, and FFN exhibited potential in maintaining PL with earlier DTH compared to the FFF combination. Similar to PL, HRP reached a plateau at approximately 88 DTH, suggesting that the FNF and FFN allele combinations may be suitable for breeding early heading rice varieties without sacrificing grain appearance. Taken together, our analyses indicate that the strategic incorporation of *hd16* (FNF) or *ghd7* (FFN) allele for breeding early maturing rice varieties may effectively reduce the growth duration without adversely affecting yield and grain quality traits such as PL and HRP.

## Discussion

### Pleiotropic effects of heading date genes on grain yield and quality

Understanding the pleiotropic effects of heading date genes on major agronomic traits is crucial for breeding rice varieties with optimal maturity and productivity for target environments. In rice, several key genes controlling heading date have been discovered to exert significant influence on yield-related traits. Notably, *Hd1*, *Hd16*. and *Ghd7* have been shown to exhibit pleiotropic effects on heading date, plant height, panicle development, and yield potential (Xue *et al*. 2008; Hori *et al*. 2012; Zhang *et al*. 2012; Hori *et al*. 2013; Weng *et al*. 2014; Chen *et al*. 2023). In this study, we detected pleiotropic QTLs colocalized with *Hd1*, *Hd16*, and *Ghd7* (Fig. 2 and Table 1), and further validated that the 3 loci exhibit significant main effects on DTH, CL, PL, and HRP (Table 2). Previous literature suggests that the pleiotropic effects of the heading date genes on CL and PL are likely genetic, given their functional involvement in internode elongation or panicle development (Endo-Higashi and Izawa 2011). However, the effects of these genes on HRP is less clear with the following possibilities to be further investigated: (1) The influence of heading date genes on HRP can potentially be attributed to “environmentally induced pleiotropy” (Auge *et al*. 2019). In temperate regions, earlier heading exposes rice plants to hot summer weather during grain filling, which typically results in deteriorated HRP (Chun *et al*. 2009; Kim *et al*. 2011; Tanamachi *et al*. 2016). (2) Alternatively, heading date genes might exhibit genetic pleiotropy on HRP as well as CL and PL, potentially through their involvement in starch accumulation during grain filling. (3) Additionally, it is plausible that there are other genes affecting HRP (and other agronomic traits) in close proximity to the heading date genes, which might have been missed due to tight linkage or the limited number of molecular markers and RILs used in our present study. Further exploration is necessary to comprehensively assess the genetic mechanisms underlying HRP and its correlation with DTH.

### Varying effects of three-gene combinations in regulating agronomic traits

Previous studies have been conducted to elucidate the genetic effects of different heading date genes and their combinations on major agronomic traits: *Hd1*/*Ghd7*/*OsPRR37* (Fujino *et al*. 2019), *Hd1*/*Ghd7* (Zhang, Zhu, Wang, *et al*. 2019), *Ghd7*/*Ghd8*/*OsPRR37*/*Hd1* (Zhang, Liu, Qi, *et al*. 2019), *Hd1*/*Ghd7*/*DTH8* (Zong *et al*. 2021; Sun *et al*. 2022), *Hd1*/*DTH8* (Fujino 2020), and *Ghd7*/*Ghd8*/*Hd1* (Zhang *et al*. 2015). In the present study, we focused on the main and combined effects of *Hd1*, *Hd16*, and *Ghd7*, the major photoperiod-sensitive genes that played significant roles in rice adaptation to diverse environments (Yano *et al*. 2000; Xue *et al*. 2008; Hori *et al*. 2013). Our study conducted under the natural long-day environments, revealed that nonfunctional *hd1* (NFF), *hd16* (FNF), and *ghd7* (FFN) could independently promote heading while concurrently decreasing CL, PL, and HRP with varying degrees. Furthermore, pyramiding 2 or more alleles for early heading (NNF, NFN, FNN, and NNN) could significantly affect DTH, CL, PL, and HRP. These results are in agreement with the previous findings that nonfunctional *hd1*, *ghd7*, and *hd16* alleles accelerate heading under long days and play important roles in rice adaptation to high latitude regions (Yano *et al*. 2000; Yano *et al*. 2001; Xue *et al*. 2008; Hori *et al*. 2013; Zhang *et al*. 2015). The present work also emphasizes the importance of understanding the interaction and integration of different heading date genes and elucidating their impacts on key agronomic traits. This understanding holds paramount importance in breeding early maturing varieties, ensuring the concurrent maintenance of grain yield and quality.

### Optimizing heading date to maximize yield

Heading date optimization represents a crucial objective in rice breeding as it aims to enhance land use efficiency by facilitating diverse double cropping systems involving rice and alternative crops. Additionally, this optimization is integral for ensuring consistent yield and superior grain quality in specific target environments (Takahashi *et al*. 2009; Fujino *et al*. 2010; Ebana *et al*. 2011). A particular focus lies in the development of early heading rice cultivars, as they play a pivotal role in regions with high latitudes, where early heading characteristics are necessary for rice plants to complete grain filling before the onset of cold winter conditions (Izawa 2007b; Shrestha *et al*. 2014). These early heading rice cultivars prove invaluable in confronting the challenges posed by climate change, allowing for better adaptability to extreme weather events and aiding in the reduction of methane emissions from rice paddies by minimizing the duration of rice cultivation (Lee *et al*. 2012; Korea 2020; Lee *et al*. 2023).

To emphasize, a longer growth duration due to delayed heading days does not always result in a higher yield (Zhang, Zhu, Wang, *et al*. 2019). When compared to the FFF group, which exhibited 22.1 cm PL, the NFF, FNF, and FFN groups with earlier heading exhibited PL of 22.7 cm, 22.5 cm, and 22.4 cm, respectively (Fig. 4). As for HRP, the FFF group showed 66.7%, while the FNF and FFN groups exhibited 65.2 and 67.8%, respectively. In the RIL population, these groups can be considered as the optimum allelic combinations maintaining yield and quality-related traits with reduced DTH. Our previous work showed that 111 out of 293 Korean commercial rice cultivars carry nonfunctional *hd1* alleles (Mo *et al*. 2021). However, only 7 cultivars possess Koshihikari-type *hd16*, and only 1 cultivar carries Sorachi-type *ghd7* (Mo *et al*. 2020). Since deploying a single allele of Koshihikari-type *hd16* or Sorachi-type *ghd7* presents a possibility of accelerating heading without adversely affecting PL and HRP, further development of early maturing cultivars in Korean rice breeding programs will facilitate the use of these alleles for fine-tuning flowering time without deteriorating yield and quality-related traits. Considering the varying allelic effects of heading date genes influenced by environmental conditions and genetic backgrounds (Zhang *et al*. 2017; Kim *et al*. 2018; Fujino *et al*. 2019; Zong *et al*. 2021), it is important to emphasize that optimal allelic combinations of heading date genes should be customized for distinct target environments and markets.

## Supplementary Material

jkad300_Supplementary_Data

## Data Availability

Genotypes and phenotypes of 142 RILs used for QTL analysis are in Supplementary Tables 1 and 2. Allele types of *Hd1*, *Hd16*, and *Ghd7* and phenotypes of 339 RILs are in Supplementary Table 3. Environmental conditions during the FTs are in Supplementary Table 4. List of primers used in this study is in Supplementary Table 5. Supplemental material is available at G3 online.
